# Long noncoding RNA *FER1L4* suppresses cancer cell growth by acting as a competing endogenous RNA and regulating *PTEN* expression

**DOI:** 10.1038/srep13445

**Published:** 2015-08-26

**Authors:** Tian Xia, Shengcan Chen, Zhen Jiang, Yongfu Shao, Xiaoming Jiang, Peifei Li, Bingxiu Xiao, Junming Guo

**Affiliations:** 1 Department of Biochemistry and Molecular Biology, Zhejiang Key Laboratory of Pathophysiology, Ningbo University School of Medicine, Ningbo, Zhejiang, 315211, China

## Abstract

Aberrantly expressed long noncoding RNAs (lncRNAs) are associated with various cancers. However, the roles of lncRNAs in the pathogenesis of most cancers are unclear. Here, we report that the lncRNA *FER1L4* (fer-1-like family member 4, pseudogene) acts as a competing endogenous RNA (ceRNA) to regulate the expression of *PTEN* (a well-known tumor suppressor gene) by taking up miR-106a-5p in gastric cancer. We observed that *FER1L4* was downregulated in gastric cancer and that its level corresponded with that of *PTEN* mRNA. Both *FER1L4* and *PTEN* mRNA were targets of miR-106a-5p. Further experiments demonstrated that *FER1L4* downregulation liberates miR-106a-5p and decreases the abundances of *PTEN* mRNA and protein. More importantly, *FER1L4* downregulation accelerated cell proliferation by promoting the G_0_/G_1_ to S phase transition. We conclude that one mechanism by which lncRNAs function in in tumorigenesis is as ceRNAs for tumor suppressor mRNAs.

Long noncoding RNAs (lncRNAs) regulate gene expression at many levels that include transcriptional, post-transcriptional and translational regulation^1^,^2^,^3^,^4^. Increasing numbers of studies have indicated that lncRNAs play key roles in tumorigenesis and may be used in the diagnosis of cancers^3^,^5^. Our previous study revealed that the fer-1-like family member 4, pseudogene (lncRNA-*FER1L4*) is strongly downregulated in gastric cancer^6^,^7^. However, the biological significance of this phenomenon is unknown.

Salmena and colleagues proposed a competing endogenous RNA (ceRNA) hypothesis to explain how different types of RNAs communicate with each other via microRNAs (miRNAs)^8^. According to this hypothesis, mRNAs, lncRNAs and other RNAs act as natural miRNA sponges to suppress intracellular miRNA function by using shared miRNA response elements (MREs)^9^,^10^,^11^. This hypothesis has been supported by numerous studies^12^,^13^,^14^,^15^. Because they are not actively translated, noncoding RNAs are thought to be highly effective ceRNAs^8^. Subsequent studies revealed that lncRNAs, including *linc-MD1*^15^, *lincRNA-RoR*^16^,^17^, *H19*^18^, *HOTAIR*^19^,^20^, *CARL*^21^, *lncRNA-ATB*^22^, and *lncRNA-BGL3*^23^, may function as ceRNAs.

Our previous work indicated that *FER1L4* is a target of miR-106a-5p^24^. The well-known tumor suppressor gene-phosphatase and tensin homolog (*PTEN*) mRNA is also a validated target of miR-106a-5p^12^. In this study, we investigated whether *FER1L4* can act as a ceRNA for *PTEN* mRNA through miR-106a-5p. We found that *FER1L4* acted as a ceRNA to regulate *PTEN* expression by acting as a sponge for miR-106a-5p in gastric cancer. *FER1L4* downregulation has been found to be a characteristic molecular change in gastric cancer^6^ and to lead to decreases in *PTEN* mRNA and protein levels. Because PTEN is a negative regulator of the cell cycle, we studied the effects of the downregulation of *FER1L4* on the cell cycle; downregulation of *FER1L4* by small interfering RNAs (siRNAs) increased cell proliferation by promoting the G_0_/G_1_ to S phase transition.

## Results

### *FER1L4* and *PTEN* mRNA are downregulated in gastric cancer

**Figure 1:**
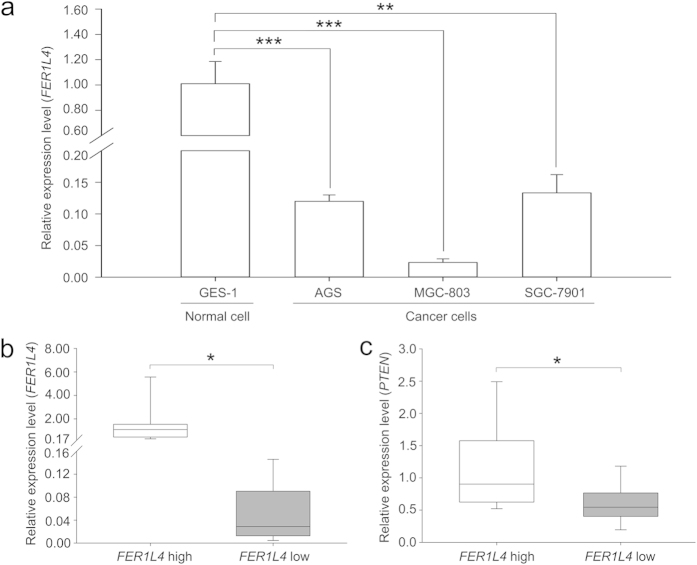
Expression of *FER1L4* in gastric cancer cells and tissues. (**a**) Expression of *FER1L4* in a human normal gastric epithelial cell line and human gastric cancer cell lines. Data are presented as mean ± SD, *n* = 3. **P < 0.01, ***P < 0.001. Expression of *FER1L4* (**b**) and *PTEN* (**c**) in the “*FER1L4* high” and “*FER1L4* low” subsets. *n* = 10, *P < 0.05.

Our previous work revealed that the *FER1L4* level is significantly lower in gastric cancer tissues than in paracancerous tissues^6^. In this study, we further found that *FER1L4* levels in the human gastric cancer cell lines AGS, MGC-803 and SGC-7901 were lower than those in the human normal gastric epithelial cell line GES-1 (Fig. 1a). These results suggest that decreases in the level of *FER1L4* may be associated with the occurrence of gastric cancer.

Considering that *FER1L4* and *PTEN* mRNA are targets of miR-106a-5p^12^,^24^ and that miR-106a-5p is highly expressed in gastric cancer^25^, we speculated that *FER1L4* and *PTEN* mRNA may act as a pair of ceRNAs that are linked by miR-106a-5p. An important corollary of the ceRNA hypothesis is that ceRNAs coordinately regulate each other’s expression^8^. Based on the above findings, we investigated whether *FER1L4* was coexpressed with *PTEN* in human samples. We measured *FER1L4* and *PTEN* expression in 20 gastric cancer tissue samples by quantitative reverse transcription-polymerase chain reaction (qRT-PCR). The samples were sorted into two subsets (*n* = 10) according to the *FER1L4* expression level (i.e., high and low *FER1L4* groups, Fig. 1b). As shown in Fig. 1c, we observed significantly higher levels of *PTEN* mRNA in the high *FER1L4* group than in the low *FER1L4* group and vice versa. This coexpression is consistent with the ceRNA hypothesis.

### *FER1L4* and *PTEN* are targeted by miR-106a-5p in gastric cells

**Figure 2:**
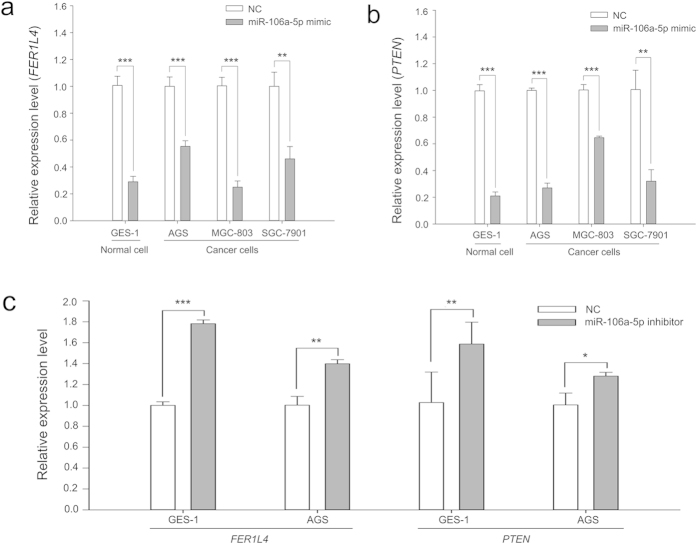
Expression of *FER1L4* and *PTEN* in a human normal gastric epithelial cell line and human gastric cancer cell lines transfected with miR-106a-5p mimics (a,b) or inhibitors (c). Data are presented as mean ± SD, *n* = 3. NC, negative control. *P < 0.05, **P < 0.01, ***P < 0.001.

*PTEN* mRNA is one of the validated targets of miR-106a-5p^12^. In our previous study, the interaction between *FER1L4* and miR-106a-5p was first predicted by miRcode and then confirmed by dual luciferase reporter assays^24^. However, we do not know whether miR-106a-5p effectively regulates *FER1L4* and *PTEN* in gastric cells. To increase the miR-106a-5p level, we transfected miR-106a-5p mimics into the normal human gastric epithelial cell line GES-1 and the human gastric cancer cell lines, AGS, MGC-803 and SGC-7901. Next, we utilized qRT-PCR analysis to reveal that miR-106a-5p suppressed both *FER1L4* and *PTEN* mRNA abundance in all of the tested gastric cell lines (Fig. 2a,b).

Moreover, to further test whether *FER1L4* and *PTEN* expression levels were linked by miR-106a-5p, we decreased the miR-106a-5p level by transfection of its inhibitors into GES-1 and AGS cells. qRT-PCR analyses indicated that the transfection of miR-106a-5p inhibitors not only increased *FER1L4* levels but also increased *PTEN* levels in both GES-1 and AGS cells (Fig. 2c). Because *FER1L4* and *PTEN* mRNA are targets of miR-106a-5p, the knockdown of miR-106a-5p led to increases in free *FER1L4* and *PTEN* mRNA.

Our previous study showed that by decreasing the expression of cyclin-dependent kinase (CDK) 1 and CDK2, the miR-106a inhibitors arrested gastric cancer cells at the G_0_/G_1_ and G_2_/M phases and then suppressed cell proliferation; and the results of animal experiments showed that the miR-106a inhibitors significantly suppressed tumor growth in a dose-dependent manner^26^. As a result, we assumed that the tumor suppression effect of miR-106a inhibitors might indirectly through the overexpression of *FER1L4*.

### Effects of *FER1L4* downregulation on miR-106a-5p and *PTEN* expression

**Figure 3:**
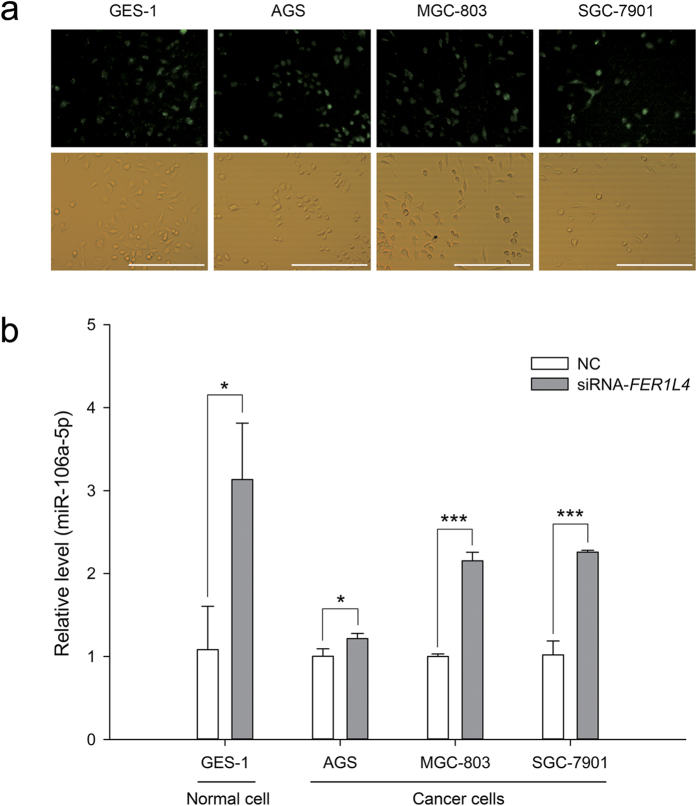
miR-106a-5p levels in a human normal gastric epithelial cell line and human gastric cancer cell lines after *FER1L4* knockdown. (**a**) transfection efficiency. Scale bars, 500 μm. (**b**) Data are presented as mean ± SD, *n* = 3. NC, negative control. *P < 0.05, ***P < 0.001.

The ceRNA hypothesis proposes that the downregulation of miRNA targets will result in freeing of the same miRNA molecules^8^. For example, GAS5-siRNA significantly reduces the endogenous GAS5 level while simultaneously increasing the miR-21 level^27^. Thus, we sought to determine whether the downregulation of *FER1L4* would influence miR-106a-5p and the levels of its targets. In our previous study, we designed a siRNA against *FER1L4* and effectively reduced *FER1L4* levels in the normal human gastric epithelial cell line GES-1 and the human gastric cancer cell lines AGS, MGC-803 and SGC-7901^24^. Here, we transfected *FER1L4*-siRNA into GES-1, AGS, MGC-803 and SGC-7901 cells, and qRT-PCR analyses revealed that the miR-106a-5p levels in the gastric cells were increased by the knockdown of *FER1L4* (Fig. 3).

**Figure 4:**
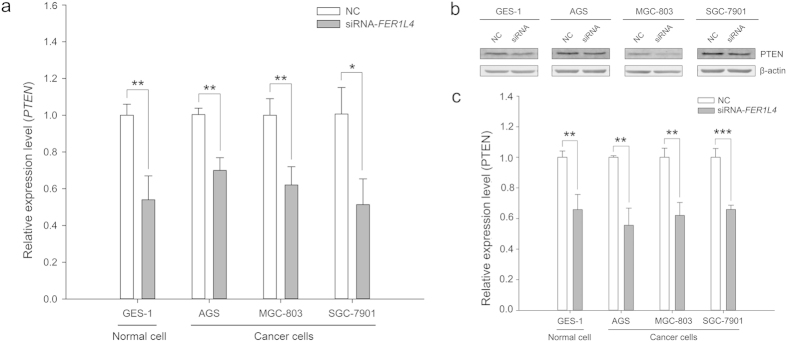
Expression of *PTEN* in a human normal gastric epithelial cell line and human gastric cancer cell lines after *FER1L4* knockdown. (**a**) *PTEN* mRNA levels detected by qRT-PCR. Data are presented as mean ± SD, *n* = 3. NC, negative control. *P < 0.05, **P < 0.01. (**b**) Representative cropped results of Western blot analyses. (**c**) Results of Western blot analyses of three independent experiments. NC, negative control. **P < 0.01, ***P < 0.001. The gels were run under the same experimental conditions. The blots were processed in parallel.

If *FER1L4* functions as a ceRNA, its downregulation might free additional miR-106a-5p. This miR-106a-5p would target *PTEN* mRNA and trigger the downregulation of *PTEN*. We knocked down *FER1L4* in GES-1, AGS, MGC-803 and SGC-7901 cells and monitored *PTEN* expression by qRT-PCR and Western blot. *FER1L4* knockdown resulted in decreased levels of *PTEN* mRNA and protein (Fig. 4).

### *FER1L4* regulates the cell cycle and cell proliferation

**Figure 5:**
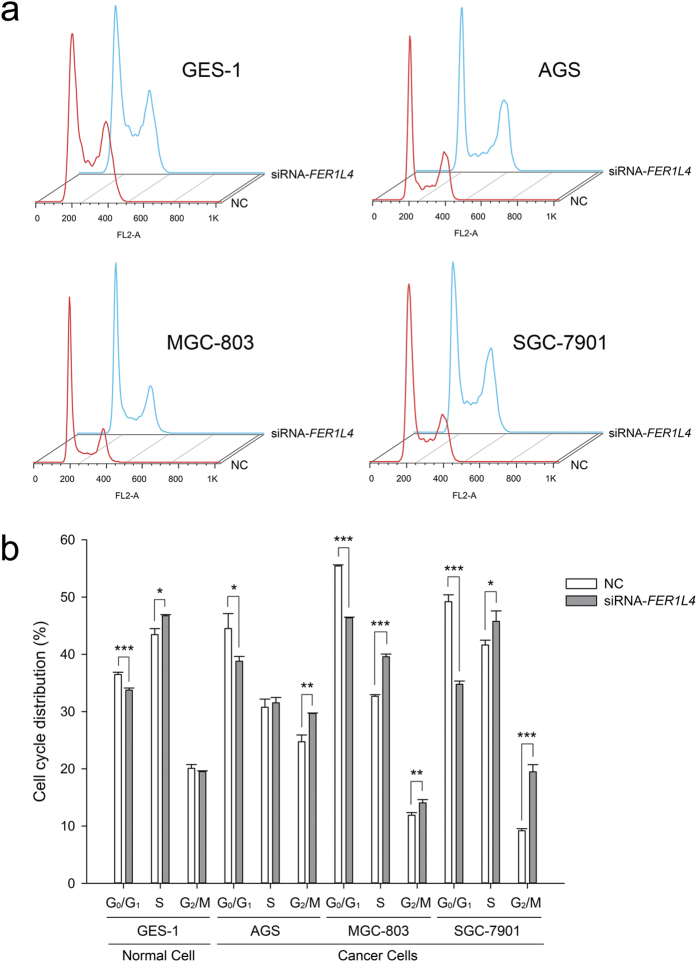
Cell cycle distributions in a human normal gastric epithelial cell line and human gastric cancer cell lines following *FER1L4* knockdown. (**a**) Representative original flow cytometry results. (**b**) Data are presented as mean ± SD, *n* = 3. NC, negative control. *P < 0.05, **P < 0.01, ***P < 0.001.

**Figure 6:**
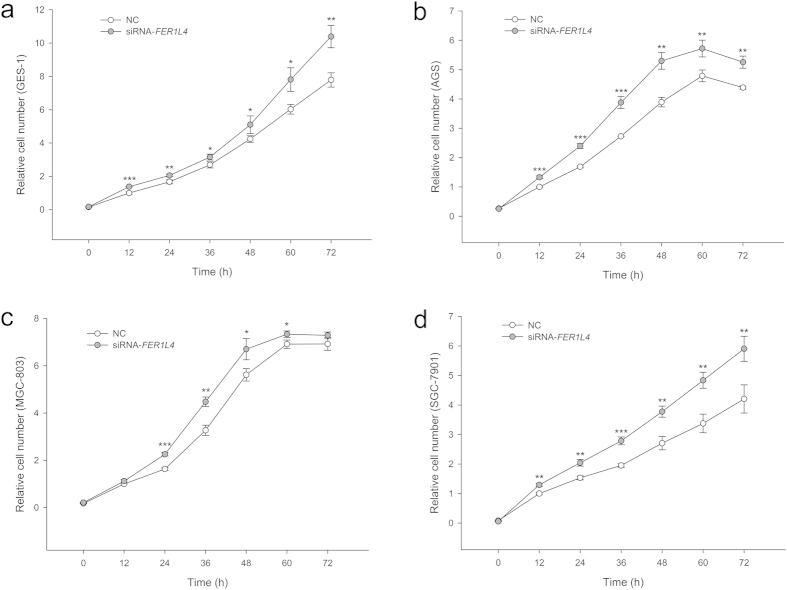
Growth curves of the human normal gastric epithelial cell line GES-1 (a) and the human gastric cancer cell lines AGS (b), MGC-803 (c) and SGC-7901 (d) following *FER1L4* knockdown. Data are presented as mean ± SD, *n* = 3. NC, negative control. *P < 0.05, **P < 0.01, ***P < 0.001.

PTEN acts as a negative regulator of the cell cycle by suppressing the phosphoinositide 3-kinase (PI3K)-AKT pathway^28^. Because perturbations of the *FER1L4* level markedly affected *PTEN* expression, we decided to investigate the effects of disrupting *FER1L4* on the cell cycle and cell proliferation. Flow cytometry demonstrated that *FER1L4* downregulation promoted the G_0_/G_1_ to S phase transition in GES-1, AGS, MGC-803 and SGC-7901 cells (Fig. 5). Moreover, *FER1L4* knockdown also accelerated cell proliferation in all of the gastric cell lines (Fig. 6).

## Discussion

Studies have revealed that ceRNAs play important roles in post-transcriptional regulation and are involved in oncogenesis and cancer progression^12^,^13^,^17^,^19^,^20^,^23^,^29^,^30^,^31^,^32^,^33^. Our previous study found that several lncRNAs may be associated with gastric cancer via their actions as ceRNAs^24^. In the present study, we focused on *FER1L4* and *PTEN* mRNA because both of these RNAs are targets of miR-106a-5p, a typical onco-miRNA^12^,^24^,^25^. *FER1L4* was downregulated in gastric cancer tissues^6^ and gastric cancer cells (Fig. 1a). *FER1L4* was coexpressed with *PTEN* such that the upregulation of *FER1L4* led to greater expression of *PTEN* and vice versa (Fig. 1b,c). To test whether *FER1L4* downregulation resulted in a reduction in the level of *PTEN* expression by freeing miR-106a-5p, we knocked down *FER1L4*. This knockdown resulted in an increase in miR-106a-5p (Fig. 3) and a decrease in *PTEN* mRNA and protein levels (Fig. 4). Furthermore, *FER1L4* downregulation accelerated cell proliferation (Fig. 6) by promoting the G_0_/G_1_ to S phase transition (Fig. 5). These results indicate that *FER1L4 and PTEN* mRNA are a pair of ceRNAs that are linked by miR-106a-5p.

**Figure 7:**
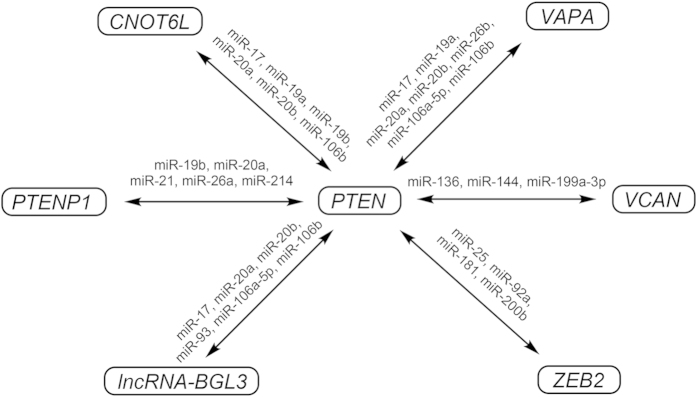
ceRNA networks associated with *PTEN*.

Indeed, the *FER1L4*-*PTEN* interaction may be associated with additional miRNAs. *FER1L4* and *PTEN* share 33 types of MREs as predicted by miRcode^34^. It has been found that several transcripts, such as *PTENP1*^29^,^35^, *CNOT6L*^12^, *VAPA*^12^, *VCAN*^30^, *ZEB2*^13^, and *lncRNA-BGL3*^23^, act as ceRNAs of *PTEN* mRNA that are mediated by pools of miRNAs (Fig. 7). These transcripts share different types of MREs with *PTEN* mRNA and are connected in a complex ceRNA network. Furthermore, these transcripts may also be ceRNAs of other RNAs with which they share different MREs. For example, *VCAN* acts as a ceRNA in the regulation of not only *PTEN* but also *RB1* and *CD34*^30^,^36^. ceRNAs may contain various MREs, and each miRNA may target hundreds of transcripts. Different pathways may interact with each other via ceRNA cross-talk^14^. In addition, whether ceRNAs can alter miRNA function *in vivo* requires further investigation^37^,^38^.

The study of ceRNA interactions represents a new approach to examining complex post-transcriptional regulatory networks. Cancer is caused by alterations in various pathways. We could design specific ceRNAs to alter these pathways for therapeutic purposes.

Recently, circular RNAs (circRNAs) were included as new members of ceRNAs^39^,^40^,^41^,^42^,^43^. A circRNA named *CDR1as* that contains approximately 70 MREs functions as a ceRNA that sequesters miR-7 away from its targets^44^,^45^. The ceRNA world is becoming increasingly more complex and fascinating. To better understand ceRNAs systematically, several groups have developed *in silico* mathematical ceRNA models to describe the characteristics of ceRNAs and ceRNA networks^46^,^47^,^48^,^49^,^50^. Moreover, several algorithms and databases, such as Linc2GO^51^, starBase^52^, ln*Ce*DB^53^, and Cupid^54^, have been developed to predict ceRNA interactions. These tools facilitate ceRNA studies.

ceRNAs can not only be used to explain biological phenomena, such as autophagy, apoptosis, and morphogenesis^55^,^56^,^57^, but can also be developed as miRNA inhibitors, such as short tandem target mimic (STTM)^58^ and circular miRNA sponges^59^. For example, circular miRNA sponges have been found to exhibit excellent anticancer effects^59^. These findings indicate that ceRNAs might represent new therapeutic approaches to cancer and other diseases^60^. Further investigations may help us to understand how ceRNAs contribute to oncogenesis and tumor metastasis. ceRNA disturbances may be associated with many diseases^61^,^62^,^63^,^64^.

In conclusion, we found that *FER1L4* was minimally expressed in gastric cancer. Via its functions as a ceRNA, *FER1L4* liberated miR-106a-5p, downregulated *PTEN* expression, and affected cell growth.

## Methods

The methods were carried out in accordance with the approved guidelines.

All experimental protocols were approved by The Human Research Ethics Committee from Ningbo University.

### Tissue samples

Biopsy samples of gastric cancer tissues were obtained at the Yinzhou Hospital Affiliated to Ningbo University School of Medicine from patients who had not undergone previous radiotherapy or chemotherapy. The Human Research Ethics Committee of Ningbo University approved all aspects of the protocols. Written informed consent was obtained from all of the subjects.

### Cells and culture conditions

The human gastric epithelial cell line GES-1 was obtained from the Cancer Institute and Hospital of the Chinese Academy of Medical Sciences (Beijing, China). The human gastric cancer cell lines, AGS, MGC-803 and SGC-7901 were obtained from the Shanghai Institutes for Biological Sciences, Chinese Academy of Sciences (Shanghai, China). All cell lines were grown in RPMI Medium 1640 (Life Technologies, Carlsbad, CA, USA) plus 10% fetal bovine serum (FBS) at 37 °C in a humidified atmosphere with 5% CO_2_. The cells were counted using a TC10 Automated Cell Counter (Bio-Rad, Hercules, CA, USA).

### Transient transfection

For the transfection of the miRNA mimics and siRNAs, GES-1, AGS, MGC-803 and SGC-7901 cells (2 × 10^5^) were seeded in 6-well plates. The following day, they were transfected with 120 nM of miRNA mimic or siRNA using Lipofectamine 2000 Reagent (Life Technologies). The sequence of the miR-106a-5p mimic was 5′-AAAAGUGCUUACAGUGCAGGUAG-3′. The sequence of the miR-106a-5p inhibitor was 5′-CUACCUGCACUGUAAGCACUUUU-3′. The sequence of the negative control was 5′-CAGUACUUUUGUGUAGUACAA-3′. The sequence of the *FER1L4* siRNA was 5′-CAGGACAGCUUCGAGUUAATT-3′ (sense) and 5′-UUAACUCGAAGCUGUCCUGTT-3′ (antisense). The sequences of the negative control siRNAs were 5′-UUCUCCGAACGUGUCACGUTT-3′ (sense) and 5′-ACGUGACACGUUCGGAGAATT-3′ (antisense). These sequences were synthesized by GenePharma Co., Ltd. (Shanghai, China).

### RNA extraction

Total RNA was extracted using TRIzol Reagent (Life Technologies) according to the manufacturer’s protocol. The RNA quantity was measured with a SmartSpec Plus spectrophotometer (Bio-Rad). The RNA purity was evaluated according to the *A*_260_/*A*_280_ ratio.

### qRT-PCR analysis

qRT-PCRs of the lncRNAs and mRNAs were performed using a GoTaq 2-Step RT-qPCR System (Promega, Madison, WI, USA) in a Mx3005P QPCR System (Stratagene, La Jolla, CA, USA) according to the manufacturer’s protocol. Glyceraldehyde-3-phosphate dehydrogenase (*GAPDH*) was used as a control. The *FER1L4* primers were as follows: forward, 5′-CCGTGTTGAGGTGCTGTTC-3′; reverse, 5′-GGCAAGTCCACTGTCAGATG-3′. The *PTEN* primers were as follows: forward, 5′-GTTTACCGGCAGCATCAAAT-3′; reverse, 5′-CCCCCACTTTAGTGCACAGT-3′. The *GAPDH* primers were as follows: forward, 5′-AAGGTGAAGGTCGGAGTCAA-3′; reverse, 5′-AATGAAGGGGTCATTGATGG-3′.

qRT-PCR of the mature miRNAs was performed using miScript II RT Kits (Qiagen, Hilden, Germany), miScript SYBR Green PCR Kits (Qiagen) and miScript Primer Assays (miR-106a-5p primer and RNU6-2 primer; Qiagen) in the Mx3005P QPCR System (Stratagene) according to the manufacturer’s protocol. RNU6-2 was used as a control.

All experiments were performed in triplicate. Relative quantification of gene expression was performed by the 2^−ΔΔ*C*t^ method^65^,^66^.

### Western blot

The cells were collected and lysed with cell lysis buffer for Western blotting (Beyotime, Haimen, China). The proteins (30 μg per lane) were separated on 12% SDS-polyacrylamide gels and transferred onto polyvinylidene fluoride (PVDF) membranes (Millipore, Billerica, MA, USA). Immunoblotting of the membranes was performed using the following primary antibodies: anti-PTEN (CST, Danvers, MA, USA) and anti-β-actin (4A Biotech, Beijing, China). The signals were revealed after incubation with the recommended secondary antibodies using an Odyssey Infrared Imaging System (LI-COR, Lincoln, NE, USA). β-actin was used as the control.

### Cell cycle analysis

The cells were washed in PBS and fixed in 75% ice-cold ethanol at − 20 °C overnight. After rehydrating with ice-cold PBS, the cells were stained with PI/RNase Staining Buffer (BD Biosciences, San Jose, CA, USA) and analyzed by flow cytometry on a FACSCalibur Flow Cytometer (BD Biosciences) using CellQuest Pro software.

### Cell proliferation assays

The proliferation assays were performed in E-Plate 96 using a Real-Time Cell Analyzer (RTCA) (ACEA Biosciences, San Diego, CA, USA) according to the manufacturer’s protocol.

### Statistical analysis

The data are presented as the means ± the SDs. The differences between groups were evaluated with two-tailed Student’s *t*-tests using SPSS Statistics 20.0 software (IBM, Armonk, NY, USA). *P* < 0.05 was considered to be statistically significant.

## Additional Information

**How to cite this article**: Xia, T. *et al.* Long noncoding RNA *FER1L4* suppresses cancer cell growth by acting as a competing endogenous RNA and regulating *PTEN* expression. *Sci. Rep.* **5**, 13445; doi: 10.1038/srep13445 (2015).
